# Nanoparticles for Ferroptosis Therapy in Cancer

**DOI:** 10.3390/pharmaceutics13111785

**Published:** 2021-10-25

**Authors:** Nadia Zaffaroni, Giovanni Luca Beretta

**Affiliations:** Molecular Pharmacology Unit, Department of Applied Research and Technological Development, Fondazione IRCCS Istituto Nazionale dei Tumori, 20133 Milan, Italy; nadia.zaffaroni@istitutotumori.mi.it

**Keywords:** ferroptosis, nanoparticles, cancer, tumor targeting, iron

## Abstract

Ferroptosis is a regulated cell death mechanism holding promise for anticancer therapy. Numerous small molecules inducing ferroptosis have been reported thus far. However, these compounds suffer from important drawbacks including poor solubility, systemic toxicity, and scarce tumor targeting ability that have limited their clinical success. The notion that nanoparticles inducing ferroptosis show better preclinical profiles compared to small molecules and overcome resistance to apoptosis has opened a new scenario for cancer treatment. Due to peculiar chemical-physical properties, nanoparticles can be loaded with anticancer drugs or decorated with tumor-selecting molecules. These features allow for drug combination treatment as well as tumor targeting. In the review, we summarize and discuss the available information concerning nanoparticles inducing ferroptosis endowed with different peculiarities and suitable for therapeutic purposes including nanoparticles for (i) antitumor drug delivery, (ii) tumor targeting, (iii) immunomodulation, and (iv) radiofrequency ablation, hyperthermia, and photodynamic therapy.

## 1. Introduction

Ferroptosis (FERR) is a regulated cell death unlike other known cell death mechanisms including necrosis, autophagy, and apoptosis [1]. Cells undergoing FERR are rounded and detached, nuclei are normal and chromatin uncondensed, mitochondrial volume and cristae are reduced, while increased mitochondrial membrane density is observed [1,2]. Aside from morphological alterations, changes in biochemical properties and gene expression are reported. Biochemical modifications include the increase in iron, lipid peroxidation, and reactive oxygen species (ROS) cellular content, reduced glutathione (GSH), and glutathione peroxidase 4 (GPX4) levels as well as solute carrier family 7 member 11 (SLC7A11) downregulation. Six genes are observed modulated in ferroptotic cells including ATP synthase F0 complex subunit C3, citrate synthase, tetratricopeptide repeat domain 35, acyl-CoA synthetase family member 2, ribosomal protein L8, and iron response element binding protein 2 (IREB2) [1]. These features are peculiar of FERR and are not observed in autophagic, necrotic, or apoptotic cells. Therefore, FERR pathways are not affected by inhibitors of autophagy, apoptosis, and necrosis [1,3].

Apoptosis has been widely studied in the past and numerous drugs acting by inducing apoptosis have successfully reached clinical use. However, resistance to apoptosis developed by tumors is responsible for treatment failure and patient death. Tumor cells resistant to apoptosis because of defects in apoptotic factors (e.g., Bax, Bak, mixed lineage kinase domain such as pseudokinase, and receptor-interacting serine/threonine-protein kinase 1/3) preserve sensitivity to compounds that induce FERR [4,5]. This feature has paved the way to use FERR inducers for counteracting and reversing the multidrug resistance to conventional anti-tumor therapies [1,6,7]. With this premise, it is evident that the discovery of agents that induce FERR is a booming wave in the field of medicinal chemistry and several compounds of this class have already been reported [8]. However, as for conventional antitumor drugs (i.e., camptothecins, platinum compounds, anthracycline, and taxanes), small molecules that induce FERR suffers from important drawbacks including poor solubility, narrow therapeutic index as well as off-target side effects [9,10]. These constraints have been faced through different biomedical approaches including the use of nanoparticles (NPs), among which NPs inducing FERR are very appealing. In addition, due to their peculiar chemical-physical properties, NPs can be loaded with antitumor drugs or decorated with tumor-targeting molecules. These features, which have proven efficacy in overcoming the drawbacks typical of small molecules, allow for drug combination treatments (e.g., induction of apoptosis and FERR in a single administration) as well as tumor targeting, thus improving the available clinical armamentarium.

In this review, we summarize the new approaches pursued in engineering NPs eliciting FERR for cancer therapy.

## 2. Cellular Mechanisms of Ferroptosis: A General Overview

**Figure 1:**
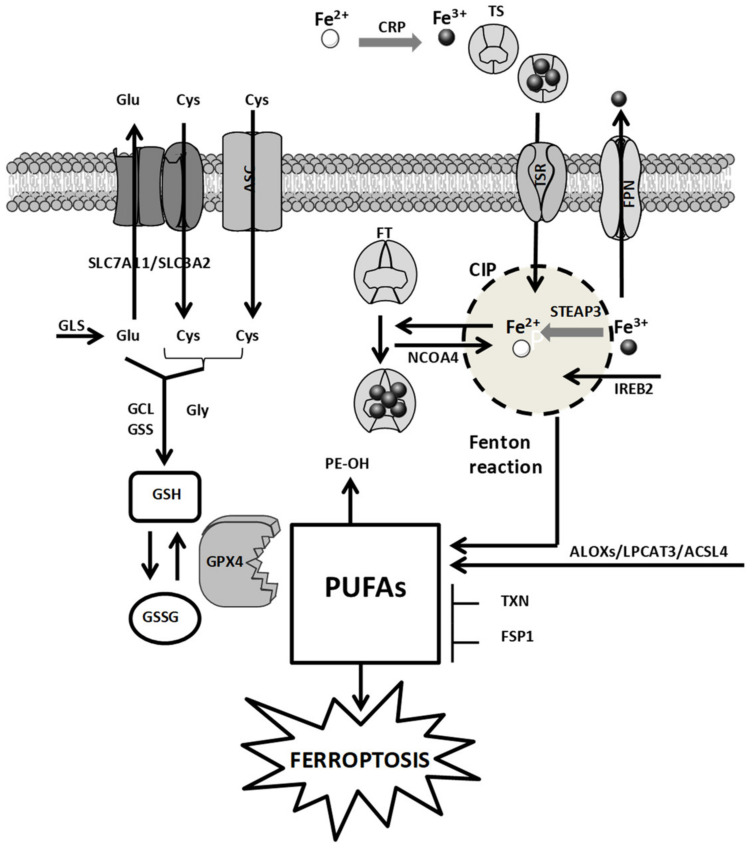
Cellular mechanisms of ferroptosis. The metabolic pathways involving iron, ROS, and amino acids are reported. CIP: cellular iron lipid; FT: ferritin; CRP: ceruloplasmin; TS: transferrin; TSR: transferrin receptor; FPN: ferroprotein; ACSL4: acyl-CoA synthetase long-chain family member 4; LPCAT3: lysophosphatidylcholine acyltransferase 3; ALOXs: arachidonate lipoxygenase; GPX4: glutathione peroxidase 4; ASC: alanine–serine–cysteine system; SLC7A11: solute carrier family 7 member 11; SLC3A2: solute carrier family 3 member 2; GLS: glutaminases; GSS: glutathione synthetase; GCL: glutamate-cysteine ligase; NCOA4: nuclear receptor coactivator 4; PUFAs: polyunsaturated fatty acids; FSP1: ferroptosis suppressor protein 1; TXN: thioredoxin; IREB2: iron response element binding protein 2. The figure was prepared using tools from Servier Medical Art (http://www.servier.fr/servier-medical-art, accessed on 15 September 2021).

The cellular mechanisms of FERR involve (i) iron metabolism, (ii) amino acid metabolism, and (iii) ROS metabolism (Figure 1) [11].

### 2.1. Iron Metabolism

Intracellular iron governs critical steps of cellular metabolism and proliferation. Because of their increased proliferation rate, cancer cells develop a sort of dependency toward iron (iron addiction). This feature over-stimulates the Fenton reaction allowing for H_2_O_2_ and ROS increase, leading to ROS-mediated biomacromolecule damage (e.g., DNA damage and lipid peroxidation) as well as the generation of a hyperoxydative status. To counteract this condition, cancer cells enhance DNA damage repair mechanisms and the levels of antioxidants, mostly GSH, also developing a GSH addiction. The cellular tolerance toward the biomacromolecule damage as well as the increased ROS levels, namely redox adaptation, renders tumor tissues more sensitive to iron-induced stress compared to their normal counterparts. This implies that in tumor cells, the iron content is finely controlled [12]. The intracellular iron level is regulated by iron transport systems including ceruloplasmin (CRP), transferrin (TS), transferrin receptor (TSR), ferritin (FT), and ferroportin (FPN). The expression of these transporters impacts the FERR proneness of tumors. CRP captures and oxidizes Fe^2+^ to Fe^3+^, which is loaded on TS and, upon interaction of TS with TSR, is taken up by the cells. Once in the cytoplasm, Fe^3+^ is reduced by the six-transmembrane epithelial antigen of the prostate 3 (STEAP3) and in this form is stored up into the cellular iron pool (CIP) or bound to FT. The overabundance of Fe^3+^ is extruded via FPN [13]. Other key regulators of CIP are the nuclear receptor coactivator 4 (NCOA4), which stimulates the accumulation of FT inside the autophagosome [14,15], the IREB2, which regulates the expression of FT [1], and the RAS oncogene, which favors iron accumulation by increasing TSR and reducing FT through the activation of the RAS–RAF–MEK pathway [16]. The interference with these pathways increases intracellular iron levels and induces FERR [3,16].

### 2.2. Amino Acid Metabolism

The redox status of the extracellular compartment controls the cystine/cystathionine exchanges across plasma membranes. The alanine–serine–cysteine (ASC) system mediates the influx of cysteine under reducing extracellular conditions. Conversely, in oxidative extracellular milieu, the cellular accumulation of cystine/cystathionine is guaranteed by the X_c_^−^ transporter system [17]. This transporter is a heterodimer containing a regulatory subunit solute carrier family 3 member 2 (SLC3A2) linked to the catalytic subunit SLC7A11 through a disulfide bridge. Since the influx of cystine/cystathionine is coupled with the efflux of glutamate, X_c_^−^ activity depends on the intracellular levels of glutamate, which in turn is regulated by the catalytic activity of glutaminases (GLS) 1 and 2. These two key enzymes of the glutaminolysis pathway are found increased in tumor tissues [18,19]. Another key regulator of FERR is the antioxidant GSH and depletion of the intracellular levels of GSH triggers FERR [20]. Two enzymatic reactions catalyzed by glutathione synthetase (GSS) and glutamate-cysteine ligase (GCL) regulate the cellular levels of GSH. Since these enzymes require glutamate, cysteine, and glycine, X_c_^−^ activity influences GSH levels. These pathways regulate GSH content and GPX4 activity, thereby impacting on polyunsaturated fatty acid (PUFA) cellular content, lipid peroxidation, and FERR. Compounds that inhibit X_c_^−^ functions (e.g., erastin) as well as molecules that impair GSH biosynthesis (e.g., buthionine sulfoximine) induce FERR by causing GSH depletion.

The transcriptional functions of p53 also regulate FERR. GLS2, SLC7A11, and spermidine/spermine N1 acetyltransferase 1 (SAT1) are controlled by p53. p53-mediated altered expression of these factors impairs cysteine uptake and stimulates lipid peroxidation and FERR [21,22,23,24]. Conversely, the p53–p21 axis increases the levels of GSH through CDKN1A activation, leading to FERR inhibition [25].

The expression of the efflux transporter multidrug resistance protein 1 (MRP1), which is implicated in tumor resistance to chemotherapy, is also involved in the regulation of the intracellular GSH levels. GSH inhibitors sensitize cancer cells, overexpressing MRP1 to FERR [26].

### 2.3. ROS Metabolism

The class of ROS comprises a series of molecules containing instable oxygen including peroxides (H_2_O_2_), singlet oxygen (^1^O_2_), radicals (HO^•^, HO_2_^•^, R^•^, RO^•^, NO^•^, and NO_2_^•^), and superoxide (O_2_^−•^). The cellular accumulation of ROS favors FERR by inducing nucleic acid, protein, and lipid damages [27,28]. PUFAs are very susceptible to lipid peroxidation, and cells showing high levels of PUFAs are prone to FERR [29,30]. Two enzymes that regulate the incorporation of PUFAs into membrane phospholipids including acyl-CoA synthetase long-chain family member 4 (ACSL4) and lysophosphatidylcholine acyltransferase 3 (LPCAT3), sensitize cells to FERR. Therefore, the propagation cycle, which favors the decomposition of lipid peroxides triggering the formation of additional ROS potentiates FERR induction [31,32].

Another factor impacting on ferroptotic cell death is the scaffold protein Raf1 kinase inhibitory protein (RKIP1). By interacting with the iron-containing enzyme arachidonate lipoxygenase 15 (ALOX15), RKIP1 favors the production of hydroperoxy polyunsaturated phospholipids leading to FERR [33].

A key factor involved in FERR is the seleno enzyme GPX4. PUFAs are metabolized by GPX4, which, in the presence of GSH, catalyzes their transformation in phospholipid alcohols, detoxifying cells from PUFAs [34]. This implies that downregulation of GPX4 expression as well as reduced enzyme activity sensitize cells to FERR [11,16]. GPX4 enzymatic activity depends on the selenocysteine tRNA, and since the mevalonate (MVA) pathway increases the levels of selenocysteine tRNA, the inhibition of this pathway negatively impacts on GPX4 activity and induces FERR [35].

Tumor cell lines resistant to FERR show high levels of ferropstosis suppressor protein 1 (FSP1). Myristoylated FSP1 anchors the cellular membrane and catalyzes the reduction of coenzyme Q10, which traps lipid peroxyl radicals, impairing the lipid peroxide propagation cycle. This behavior suppresses phospholipid peroxidation, resulting in FERR inhibition [36,37].

## 3. Nanoparticles Eliciting Ferroptosis for Cancer Therapy

**Figure 2:**
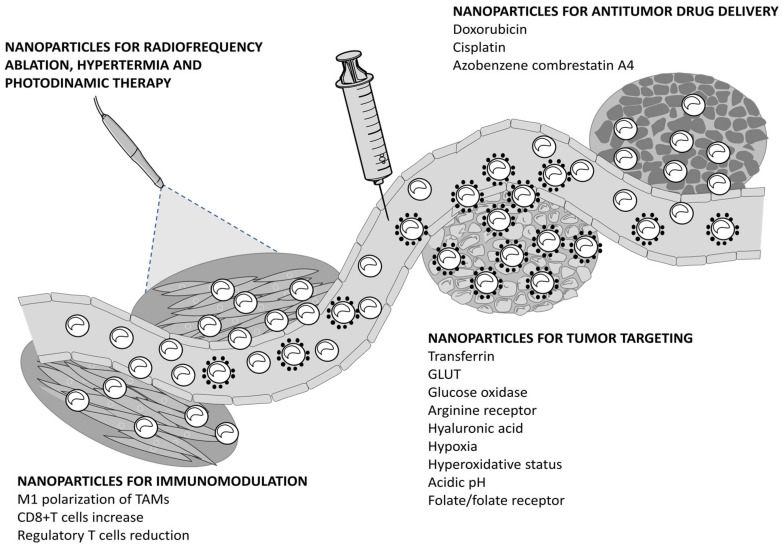
Mechanisms of action of nanoparticles inducing ferroptosis. The figure was prepared using tools from Servier Medical Art (http://www.servier.fr/servier-medical-art, accessed on 15 September 2021).

Due to increased metabolism and cell proliferation, tumors are strongly dependent on iron content and suffer from a hyperoxidative condition. Since iron is crucial for FERR induction and iron overload sensitizes tumors toward FERR, NPs capable of rapidly and massively increasing the cellular levels of iron are promising for cancer therapy. By virtue of their physical properties, these NPs exploit the enhanced permeability retention (EPR) effect to select the tumor. Additionally, their loading capability as well as the chance to selectively target tumors via molecule-specific functionalization have been shown to improve antitumor potency compared to small molecules inducing FERR (Figure 2).

### 3.1. Nanoparticles for Antitumor Drug Delivery

NPs assembling doxorubicin (DOX), tannic-acid (TA), and the photosensitizer IR820 have been reported by Xiong and coworkers (DAR, Table 1) [38]. The cellular accumulation of DAR occurs through the endocytic pathway, which leads to lysosome disruption and endogenous iron hijacking, in turn, inducing FERR. Although the NPs release DOX and IR820 in the absence of irradiation, laser exposure (808 nm) intensifies drug release and increases iron-mediated ROS production in human MCF7 breast cancer cells. Aside from inducing FERR, ROS accumulate into the endoplasmic reticulum (ER) causing ER stress (e.g., calreticulin and CHOP upregulation) and potentiate the DOX-mediated immunogenic cell death (ICD). The in vitro results are corroborated by in vivo studies performed in MCF7 and mouse mammary 4T1 tumors, which confirm the synergism between FERR and ICD mediated by DAR/laser exposure. No comparison with the antitumor potency of free DOX is reported in the studies.

A totally dissoluble amorphous calcium carbonate NPs encapsulating DOX-Fe^2+^ complex (ACC@DOX–CaSi–PAMAM–FA/mPEG) is proposed by Xue et al. (Table 1) [39]. The NPs are functionalized with folate (Fl) and a polyethylene glycol (PEG) polyamidoamine (PAMAM) dendrimer containing a matrix metalloproteinase–2 (MMP-2)–cleavable peptide that guarantees NP stability in blood circulation and tumor-specific uptake. NPs are stable in biologic fluids and release their cargo in the presence of high MMP-2 levels typical of the extracellular environment of invasive tumors. The MMP-2-mediated shedding of the dentrimer exposes Fl and promotes Fl-receptor-mediated tumor targeting. FERR (i.e., NOX4-mediated H_2_O_2_ and lipid peroxides increment) and apoptosis (i.e., caspase 3 activation and Bax upregulation as well as Bcl-2 downregulation) induction have been observed in mammary mouse 4T1 tumor cells and human A375 melanoma cells treated with ACC@DOX–CaSi–PAMAM-FA/mPEG. Furthermore, reduced tumor growth and increased mice survival have been reported in vivo in 4T1 and A375 tumor suffering mice treated with NPs.

FERR and apoptosis cell death are also reported in tumor cells treated with DGU:Fe NPs designed by Bao and colleagues (Table 1) [40]. The core of the NPs contains Fe^3+^ cross-linked with DOX through an oxidized starch sensitive to the charge conversion occurring in an acidic microenvironment. These features implement the circulation time and favor the EPR effect as well as lysosomal escape. Moreover, near infrared (NIR) irradiation enables the Fe^3+^/Fe^2+^ reduction, leading to DOX and Fe^2+^ release, which induces apoptosis and FERR, respectively. Compared to free DOX, a superior antitumor potency is reported in mice bearing 4T1 and MCF7 tumors treated with DGU:Fe.

Besides DOX, the antitumor drug cisplatin (cDDP) is also used in the design of degradable metallic NPs proposed by Chen et al. (PtH@FeP, Table 1) [41]. These NPs contain a Fe^3+^-polydopamine (FeP) core covered by a hyaluronic acid (HA)-cross-linked cDDP (PtH) shell. The antiproliferative activity of the NPs was tested in 4T1 mouse cells as well as in human HepG2 hepatocellular and ovarian A2780 carcinoma cell lines. Under laser irradiation (808 nm), NPs release iron and cDDP, which induce FERR (e.g., HO^•^ and NOXs augmentation and GPX4 downregulation) and apoptosis (DNA-Pt cross-links and p53-mediated DNA damage), respectively. Besides apoptosis, cDDP potentiates the iron-dependent FERR by inducing GSH depletion and preventing DNA damage repair. The antitumor activity of PtH@FeP in comparison with free cDDP was tested in mouse mammary 4T1 tumor. Compared to free cDDP, a superior tumor growth reduction and an increased mice survival were reported in the 4T1 mouse model treated with PtH@FeP.

cDDP is also the leader drug composing the FeGd-HN@Pt@LF/RGD2 NPs reported by Shen and coworkers (Table 1) [42]. These NPs contain a magnetic Fe_3_O_4_/Gd_2_O_3_ core loaded with cDDP and are decorated with lactoferrin (LF) and the Arg-Gly-Asp (RGD) dimer (RGD2) for selective targeting of brain tumors. A potent antiproliferative activity was observed in human U-87 MG glioblastoma cells exposed to FeGd-HN@Pt@LF/RGD2. Moreover, NPs easily cross the blood–brain barrier and through the LF and RGD2 moieties, internalize tumor cells via LF- and integrin αvβ3v-mediated endocytic pathways. Upon endosomal uptake and degradation, Fe^2+^/Fe^3+^ stimulates the Fenton reaction (i.e., H_2_O_2_ and HO^•^ production), which is further implemented and accelerated by cDDP, leading to FERR. The FeGd-HN@Pt@LF/RGD2 NPs successfully reached U-87 MG orthotopic tumors in mice and showed effective tumor growth inhibition. Thanks to intrinsic magnetic features provided by the Fe_3_O_4_/Gd_2_O_3_ core, tumor accumulation of NPs can be monitored using magnetic resonance imaging (MRI).

The microtubule inhibitor azobenzene combretastatin A4 (Azo-CA4), an interesting drug active on triple negative breast cancer (TNBC), is loaded on NPs (UCNP@LP(Azo-CA4), Table 1) sensible to NIR (980 nm) and UV (365 nm) irradiation [43]. NIR exposure induces the release of Fe^3+^ and trans-Azo-CA4, which are activated in Fe^2+^ and cis-Azo-CA4 upon UV irradiation. TNBC MDA-MB-231 cells treated with UCNP@LP(Azo-CA4) and irradiated undergo cis-Azo-CA4-mediated apoptosis (e.g., microtubule function impairment and G2/M cell-cycle arrest) as well as Fe^2+^-mediated FERR (e.g., peroxidation of tailored lipids), which significantly reduce cell proliferation. Tumor growth inhibitions as well as increased mice survival were observed in irradiated MDA-MB-231 tumor-bearing mice treated with UCNP@LP(Azo-CA4).

The capability of p53 to modulate the redox status of cancer cells was considered by Zheng and colleagues for the engineering of iron-based metal organic framework (MOF) NPs encapsulating a DNA plasmid encoding for p53 (MON-p53, Table 1) [44]. Although these NPs are not loaded with small molecules, they allow for the delivery of a drug-like nucleic acid encoding the antitumor p53 protein. The NPs reduce the cell growth of human HT1080 fibrosarcoma cells, mouse SCC-7 squamous, and mammary 4T1 tumor cell lines. Deep investigations into HT1080 cells revealed that FERR and apoptosis pathways are activated in cells treated with MON-p53. HT1080 cells exposed to MON-p53 increased p53 expression and underwent FERR (e.g., increased ROS and lipid peroxides and reduced GPX4 action via a GSH depletion mediated by the reduction of SLC7A11 levels) and apoptosis (i.e., caspase 3 cleavage). Additionally, the wound healing assay on 4T1 cells indicated that NPs delay cell migration. In vivo studies on HT1080 and 4T1 tumor-bearing mice showed that MON-p53 treatment reduced tumor growth, prolonged the life-span of mice, and decreased lung and liver metastasis.

### 3.2. Nanoparticles for Tumor Targeting

Besides passive targeting via EPR, several strategies have been considered for active targeting of tumors including the use of NPs decorated with agents identifying the molecular features of cancers or responsive to physiologic conditions peculiar to tumors (e.g., acidic pH as well as hypoxia or redox unbalance).

**Figure 3:**
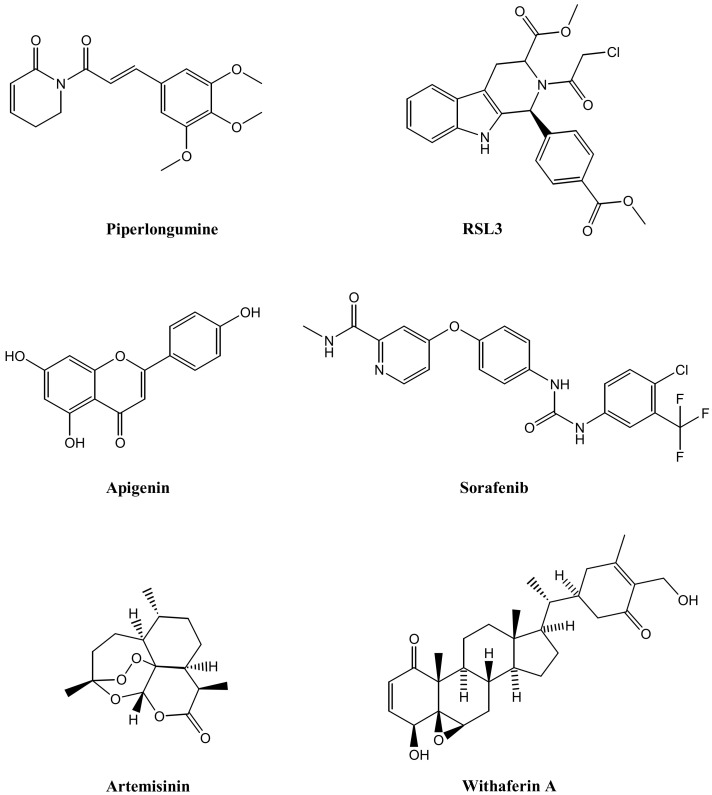
Chemical structures of small molecules inducing ferroptosis loaded on nanoparticles.

Xu et al. recently reported NPs inducing FERR decorated with TS (Table 2) [45]. The NPs were loaded with piperlongumine (FERR inducer, Figure 3) and covered with pH sensitive lipid membranes embedding TS (Tf-LipoMof@PL). In vitro studies in mouse mammary 4T1 tumor cells showed that TS facilitates the endocytic cellular uptake and enriches the intracellular iron content leading to the Fenton reaction as well as the selective delivery of piperlongumine. The latter favors ROS production, which synergizes with iron in inducing FERR. In vivo studies in 4T1 tumor suffering mice indicate that Tf-LipoMof@PL is safe and efficiently reduces tumor growth.

Glucose transporter (GLUT) was considered by Li and coworkers in the synthesis of Malt-PEG-Abz@RSL3 for the specific targeting of tumors expressing high levels of GLUT (Table 2). Malt-PEG-Abz@RSL3 are NPs containing an azobenzene moiety and a maltose ligand loaded with a small molecule inducing FERR RSL3 (Figure 3). Malt-PEG-Abz@RSL3 targets human HepG2 hepatocyte carcinoma cells expressing GLUT and allows for selective delivery of RSL3. RSL3 released by the NPs inhibits GPX4 activity, leading to FERR. Moreover, the azobenzene moiety depletes the NADPH coenzyme, which is implicated in GSH synthesis and in thioredoxin (TXN) reduction, reinforcing the RSL3-mediated FERR [46].

Starvation therapy is an anticancer strategy to sensitize tumors to drug treatment. In this context, ferric NPs (NMIL-100@GOx@C, Table 2) covered with cancer cell membranes and functionalized with glucose oxidase (GOx) have been designed [47]. The cell membrane envelop allows the immune system escape and the accumulation of the NPs into the tumors. The high GSH levels inside the tumors favor NP dissolution via Fe^3+^/Fe^2+^ reduction. Moreover, the released GOx catalyzes the oxidation of glucose, which is abundant in starved cells, implementing the intracellular amount of H_2_O_2_. The latter, together with Fe^2+^, promotes the Fenton reaction and FERR. These findings have been observed in vitro and in vivo in the mouse mammary 4T1 model.

The amino acid arginine is considered for the construction of arginine-rich manganese silicate NPs (AMSNs, Table 2) that target tumors overexpressing the arginine transporter [48]. AMSN dissolution in tumor cells releases manganese, allowing for GSH depletion and inducing FERR by inhibiting GPX4 activity. These findings were observed in vitro and in vivo in the human hepatocellular Huh 7 carcinoma model. AMSNs show an interesting DOX loading capability that potentiates their antiproliferative activity in vitro. Moreover, the presence of Mn^2+^ allows for MRI NP tracking.

Fe_2_O_3_/Fe_3_O_4_@mSiO_2_-HA are tumor targeting magnetic NPs prepared by Liu and coworkers (Table 2) [49]. These NPs are engineered for the delivery of the FERR inducer apigenin (API) and are composed of a mesoporous silica shell built on a Fe_2_O_3_/Fe_3_O_4_ core. Moreover, they are decorated with HA for tumor targeting. HA directs API-Fe_2_O_3_/Fe_3_O_4_@mSiO_2_-HA NPs toward tumors expressing high CD44 levels, which possess hyaluronidase activity. The application of a magnetic field improves the selectivity of HA-dependent cellular uptake. The NPs showed biocompatibility and sustained API release on the tumor site. Compared to the treatment with free API, enhanced ROS, superoxide dismutase, and malondialdehyde (MDA) production (e.g., FERR induction) were observed in human A549 lung carcinoma cells treated with API-Fe_2_O_3_/Fe_3_O_4_@mSiO_2_-HA.

High cellular levels of GSH and TXN detoxify from lipid peroxides and reduce ferroptotic cell death. To favor FERR induction, hypoxia-responsive NPs that selectively target hypoxic solid tumors were designed by Guo et al. [50]. These NPs are made up of a polymer containing PEG joined with nitroimidazole-conjugated polypeptide via an azobenzene linker and are loaded with the small molecules inducing FERR RSL3 (mPEG-Azo-PBLA@RSL3, Table 2 and Figure 3). Hypoxic conditions induce the disruption of the azobenzene linker, leading to PEG shedding and exposing the nitroimidazole moiety to the enzymatic activity of nitroreductase. The NADPH-mediated reduction of nitroimidazole causes a cellular depletion of NADPH, which downregulates GSH and TXN and potentiates the FERR induced by RSL3. Mouse mammary 4T1 cells treated with these NPs showed NADPH depletion as well as GSH and TXN downregulation both in vitro and in vivo.

The antioxidant GSH counteracts toxic oxidative stress stimuli favoring cell survival. Because of increased metabolism and proliferation rate, cancer cells are under a hyperoxidative status and contrast this condition by implementing GSH levels with respect to normal cells. Thus, GSH depletion, which impairs the redox balance of cancer cells, represents a strategy for tumor targeting. This feature guided Tang and colleagues in the design of manganese silica NPs (FaPEG-MnMSN@SFB) encapsulating the FERR inducer sorafenib (SFB) (Table 2 and Figure 3) [51]. Tumor cells (HepG2, 4T1, and A549) treated with FaPEG-MnMSN@SFB undergo redox alterations that cause GSH depletion. FaPEG-MnMSN@SFB dissolution releases MnMSN and SFB. MnMSN consumes GSH, while SFB inhibits the X_c_^−^ transport system. This behavior leads to GSH depletion and redox unbalance, which increases ROS, in turn, inducing apoptosis and FERR. In vivo studies performed in HepG2 xenograft-bearing mice confirmed the theranostic potential of FaPEG-MnMSN@SFB. Indeed, aside from tumor growth reduction and the improvement in mice survival, these NPs are useful for MRI due to the magnetic properties of Mn^2+^. Similarly, Zhou et al. projected NPs containing ferrocene (Fc) loaded with RSL3 (RSL3@COF-Fc) (Table 2 and Figure 3) [52]. These NPs reduce the growth of tumor cells (HT-1080, MCF-7 and HCT-116) in vitro and downregulate the enzymatic activity of GPX4. RSL3@COF-Fc NPs impair GSH synthesis and promote the Fenton reaction, which favors HO^•^ as well as lipid peroxide accumulation, leading to FERR. These findings are confirmed in the human HT-1080 fibrosarcoma xenograft mouse model. RSL3@COF-Fc NPs efficiently reduce tumor growth as well as GPX4 activity and GSH content, while increasing the MDA amounts.

Mitochondrial membrane functionalized photosensitive NPs (CSO-SS-Cy7-Hex/SPION/Srfn, Table 2) capable of targeting tumor cells undergoing epithelial–mesenchymal transition (EMT) were reported in the study by Sang and co-workers [53]. The NPs are loaded with SFB (Figure 3) and following cellular uptake, the redox response of the cell allows for the shedding of Cy7-Hex and SFB. The former favors the anchoring of NIR photosensitizer Cy7 to the mitochondrial membranes via hexadecane (Hex) arms while the latter inhibits the X_c_^−^ transport system. Upon irradiation, ROS and lipid peroxides increase and this feature allows for mitochondria collapse. The ferroptotic cell death induced by GSH depletion (e.g., reduced expression of SLC7A11 and GPX4 as well as increased lipid peroxide accumulation) is further implemented by the release of iron by SPION. These findings were observed in vitro in mouse mammary 4T1 and human MDA-MB-231 breast carcinoma cell lines. Moreover, CSO-SS-Cy7-Hex/SPION/Srfn NPs selectively target 4T1 cells undergoing EMT upon transforming growth factor β1 (TGF-β1) stimulation. Injected in mice, these NPs are well tolerated and following NIR irradiation, increase the circulating time of SFB and efficiently reduce tumor growth.

A typical feature of tumors is the acidic microenvironment and this peculiarity is considered to direct pH responsive NPs to the tumor site. Highly aggressive, poorly responding TNBC are very sensitive to redox unbalance and the induction of FERR in this tumor type proved efficacy [54]. To target TNBC, Li and colleagues proposed NPs based on TA and Fe^2+^ encapsulated into a zeolitic imidazolate framework (TA-Fe/ART@ZIF) and loaded with the FERR inducer artemisinin (ART) (Table 2 and Figure 3). These NPs are pH sensitive and trigger Fe^2+^ and ART release at pH 5.0. Increased ROS and MDA levels coupled with reduced GSH and GPX4 expression are shown in human MDA-MB-231 TNBC cells exposed to TA-Fe/ART@ZIF. Therefore, a tumor growth reduction was observed in vivo in mice bearing the MDA-MB-231 tumor xenograft treated with TA-Fe/ART@ZIF.

HA@MOF NPs sensitive to pH were engineered by Xu and coworkers [55]. These NPs are composed of ferrous acetate and benzene dicarboxylic acid ligands coated with HA (Table 2). The ligand is sensitive to pH, and under acidic conditions favors the dissolution of NPs and the release of Fe^2+^. Mouse mammary 4T1 tumor cells treated with HA@MOF increase Fe^2+^ and ROS levels, which in turn induce FERR. The in vitro results are supported by in vivo studies performed in mice suffering from mammary 4T1 tumors. The HA@MOF result is biocompatible and showed impressive inhibition in tumor growth and an important improvement in the health of treated mice.

An interesting compound active on neuroblastoma is the natural FERR-inducing agent withaferin A (WA, Figure 3) [56]. Aside from being a conventional mechanism of FERR induction (i.e., GPX4 inhibition), this compound is endowed with a non-conventional mechanism, which implies the targeting of Kelch-like ECH-associated protein 1 and the activation of the nuclear factor-like 2 pathway. The activation of the non-conventional pathway massively increases the heme oxygenase-1 activity that provokes the increase in the intracellular Fe^2+^ levels, in turn implementing the conventional FERR mechanism. The compound is more active than the conventional antitumor drugs etoposide and cDDP on a wide panel of neuroblastoma cell lines including IMR-32, SK-N-SH, Kelly, NB69, NFL, SH-EP, SH-SY5Y, SK-N-AS, SK-N-BE(2)C, SK-N-DZ, and CHP-134. However, due to its scarce solubility, mice treated with free WA manifest severe adverse side effects. To face this constraint, Hassannia and coworkers formulated WA in amphiphilic degradable pH-sensitive NPs (WA-NPs, Table 1). Systemic administration of WA-NPs in IMR-32 tumor xenograft-bearing mice reduces the side effects typical of free WA and efficiently suppresses tumor growth.

An innovative strategy proposed to selectively kill tumor cells is the use of nanozymes endowed with peroxidase-like activity. These enzymes catalyze the production of HO^•^ from H_2_O_2_ and in such a way as to induce FERR. However, the application of this strategy has shown difficulties because of the low levels of H_2_O_2_ in tumors and the scarce affinity of the nanozymes for H_2_O_2_, which results in insufficient amounts of HO^•^ to achieve tumor killing. To improve enzyme activity, a pyrite-peroxidase nanozyme NPs with high affinity for H_2_O_2_ was engineered (Table 2) [57]. In comparison to horseradish peroxidase and the conventional Fe_3_O_4_-based nanozyme, these NPs possess high peroxidase activity and catalyzes the production of H_2_O_2_ via GSH oxidation in cell-free systems. Treatment of mouse CT26 colon adenocarcinoma cells with NPs induce increased intracellular iron content, cell-cycle arrest in the G2M phase, and reduced cell proliferation via FERR (e.g., HO^•^ and lipid peroxides increase as well as GSH depletion) and apoptosis. Injected in mice bearing CT26 tumors, these NPs exhibited superior antitumor potency compared to the Fe_3_O_4_-based nanozymes. Moreover, Fl functionalized NPs showed selectivity for CT26 cells expressing high levels of the Fl receptor both in vitro and in vivo.

Other NPs based on the enzyme-mediated H_2_O_2_ production and endowed with tumor killing activity are the PEGylated single-atom Fe-containing nanocatalysts (PSAF NCs, Table 2) proposed in the study by Huo et al. [58]. Under the acidic conditions of the tumor microenvironment, these NPs stimulate the proton-mediated H_2_O_2_-homolytic pathways leading to apoptotic cell death and FERR via the accumulation of lipid peroxides, as observed in 4T1 tumor cells exposed to PSAF NCs. Moreover, PSAF NCs showed a favorable biodegradability and biocompatibility as well as tumor accumulation and antitumor activity in 4T1-tumor bearing mice.

### 3.3. Nanoparticles for Immunomodulation

Tumor-associated macrophages (TAMs) are importantly involved in tumor development/progression and the modulation of their functions (i.e., the switch M2/M1) represents an intriguing medical option. However, the polarization of TAMs toward the M1 tumor-killing type is attenuated by the release of anti-inflammatory factors as well as by altered metabolic functions of the macrophages. Among the strategies considered to promote the activation of TAMs, the use of NPs inducing FERR proved efficacy [59,60,61].

Gu et al. proposed iron-based NPs (MIL88B/RSL3) loaded with the FERR inducer RSL3 (Table 3 and Figure 3). RSL3 enhances iron-mediated lipid peroxidation in cancer cells and sensitizes macrophages to pro-inflammatory signaling. Upon NP uptake, the activation of pro-inflammatory signaling pathways drives the antitumor action of TAMs by favoring the mitochondrial metabolic switch from oxidative phosphorylation to glycolysis and polarizing TAMs toward the tumor-killing M1 phenotype [59]. This behavior impacts mouse mammary 4T1 tumor growth in vivo. Compared to the conventional lipopolysaccharides and interferon gamma (IFN-γ) therapy, MIL88B/RSL3 show superior antitumor potency.

The simultaneous targeting of cancer cells and tumor microenvironment was achieved using zero-valent-iron NPs (ZVI-NP, Table 3). Aside from hitting cancer cells by inducing iron-mediated FERR, these NPs stimulate anticancer immunity by reprogramming the microenvironment [60]. Human (A549, H460, H1299) and mouse (LLC) lung carcinoma cells exposed to ZVI-NP showed alterations in redox balance, mitochondria dysfunction, and lipid peroxidation. These features induce FERR through the ubiquitination-dependent degradation of the nuclear factor-E2-related factor 2 mediated by the activation of the AMPK/mTOR–GSK3/β–TrCP axis. Moreover, these NPs suppress the expression of cancer cell stemness (OCT4, Nanog, SOX2) and pro-angiogenic (TGF-β, sonic hedgehog and vascular endothelial growth factor) genes, and potentiate the anti-tumor immunity by favoring the switch of TAMs toward M1 and increasing cytotoxic CD8+ T cells, while reducing regulatory T cell levels.

Other interesting NPs endowed with immunomodulatory properties are the magnetosomes Pa-M/Ti-NC (Table 3) reported by Zhang and colleagues [61]. These NPs are made up of a magnetic Fe_3_O_4_ core covered by leukocyte membranes decorated with the PD-1 antibody and TGF-β inhibitor embedded into the lipid bi-layer. Following intravenous administration and magnetization, Pa-M/Ti-NC NPs target the tumor and enable MRI. The TGF-β inhibitor and PD-1 antibody generate an immunogenic microenvironment that increases the production of H_2_O_2_ and HO^•^, which induces FERR in tumor cells and favors TAMs polarization toward M1. In vivo experiments performed in mice suffering from mammary 4T1 tumors showed that Pa-M/Ti-NC administration reduced tumor growth and lung metastasis and improved mice survival.

### 3.4. Nanoparticles for Radiofrequency Ablation, Hyperthermia, and Photodynamic Therapy

Radiofrequency ablation (RFA) is clinically used to fight solid tumors. This strategy is ineffective on metastasis and rarely achieves complete tumor eradication. The CaCO_3_ HLCaP NPs encapsulating lipoxidase and hemin with poly-lactic-co-glycolic acid (PLGA) are projected to improve RFA (Table 4) [62]. Profiting by the cancer cell debris as a fuel, HLCaP produce lipid radicals through the lipid peroxidation chain reaction, and in such a way, provoke FERR-mediated cell cytotoxicity. These findings have been corroborated in vivo in mice (breast cancer 4T1 and hepatoma H22) and rabbits (hepatocellular carcinoma VX2)-bearing tumors as well as on patient-derived xenograft tumors. HLCaP NPs accumulate in residual tumors and metastasis and after RFA exposure, suppress the growth of both tumors and metastasis by triggering FERR.

The reduced levels of antioxidants observed in tumors under mild hyperthermic conditions (45 °C) impaired redox balance and increased lipid peroxides sensitizing to FERR. Xie and colleagues triggered heat-mediated FERR by using a polypeptide-modified Fe_3_O_4_-containing 1H-perfluoropentane (1H-PFP) NPs (GBP@Fe_3_O_4_, Table 4) [63]. When irradiated at 808 nm, the phase transition of 1H-PFP provokes hyperthermia, which locally releases Fe_3_O_4_ and increases ROS and lipid peroxides. The increased level of the acyl-CoA synthetase ACSBG1 observed in tumor cells exposed to these NPs drives the reprogramming of the lipid metabolism that, together with the reduction in antioxidants generated by stress-induced hyperthermia, increases FERR and promotes antitumor activity. These findings have been observed in different tumor cell lines (e.g., breast MBA-MD-231, renal 786-O, and prostate PC3 and C4-2) exposed to GBP@Fe_3_O_4_ upon irradiation, and are reversed in knock-out ACSBG1 cells. A complete tumor regression is reported in PC3 tumor-bearing mice after treatment with GBP@Fe_3_O_4_ and laser irradiation.

The combination of FERR induction and photodynamic therapy (PDT) is an intriguing option for cancer treatment. PDT antitumor activity relies on ROS-mediated apoptosis induction. Moreover, ROS augmentation depletes GSH as well as, in turn, promoting FERR. Thus, NPs projected for PDT and capable of triggering FERR hit tumor cells by inducing both apoptosis and FERR. Based on this notion, NPs composed of PLGA and loaded with Fe_3_O_4_ and chlorin E6 (Ce6) (Fe_3_O_4_-PLGA-Ce6) have been reported (Table 4) [64]. In vitro studies in mouse mammary 4T1 tumor cells show that following cellular uptake, the dissolution of Fe_3_O_4_-PLGA-Ce6 releases Ce6 and Fe^3+^/Fe^2+^. The latter stimulates the Fenton reaction and induces FERR via GSH and GPX4 downregulation as well as MDA augmentation. Additionally, under laser irradiation, NPs release Ce6, which enhances ROS accumulation, potentiating the iron-mediated FERR pathway. In vivo studies in mice suffering from 4T1 tumors show that Fe_3_O_4_-PLGA-Ce6 are biocompatible and, besides efficient tumor growth inhibition, possess MRI usefulness due to the magnetic properties of Fe_3_O_4_.

A combination of chemo-dynamic, photo-dynamic, and immune-activating therapies has recently been reported for PEG-coated ferrihydrite NPs (PEG-Fns, Table 4). PEG-Fns NPs are highly biocompatible and taken up by the tumor cells. Under blue light illumination, they release Fe^2+^, which increases ROS levels, which in turn inhibit GPX4 activity (e.g., FERR induction) and induce DNA damage (e.g., apoptosis) [65]. Furthermore, the intravenous injection of these NPs in mice bearing 4T1 tumors induces M1 TAM polarization and reduces pulmonary metastasis.

A versatile NP (PB@FePt-HA-g-PEG) for photothermal and chemo-dynamic co-therapy as well as imaging (magnetic resonance/computed tomography/photothermal imaging) was reported by Hu et al. (Table 4) [66]. Photothermal PB@FePt-HA-g-PEG is functionalized with HA for tumor targeting and loaded with the FERR inducing agent FePt. In vitro studies in human MCF7 and mouse 4T1 breast cancer cells show that upon 808 nm irradiation, the exposure to PB@FePt-HA-g-PEG increases H_2_O_2_ and HO^•^ levels and induces FERR as well as apoptosis. The NPs are very biocompatible and after intravenous injection, allow for MRI tumor detection. Moreover, upon laser irradiation, the preferential tumor accumulation of PB@FePt-HA-g-PEG efficiently reduces the 4T1 tumor growth in vivo.

PDT efficacy strongly depends on tissue oxygen content and often fails because of the hypoxic environment generated by the tumors. To potentiate PDT under hypoxic conditions, NPs endowed with both PDT and FERR functions are engineered [67]. These NPs (SRF@Hb-Ce6, Table 4) contain a hemoglobin (Hb) joined to Ce6 (PDT inducer) and is loaded with SFB (FERR inducer). Moreover, to direct drug action to tumor tissues, an MMP2-responsive peptide is introduced. Besides providing oxygen for PDT, Hb also furnishes the iron for FERR. Increased HO^•^, lipid peroxides, and MDA levels as well as reduced GSH content were observed in human HepG2, A549, and mouse 4T1 tumor cell lines exposed to SRF@Hb-Ce6 and irradiated at 660 nm. In vivo studies in mouse 4T1 tumor suffering mice showed that tumor growth inhibition mediated by PDT after SRF@Hb-Ce6 exposure is potentiated by FERR and by the recruitment of immune cells secreting IFN-γ.

Other NPs combining PDT and FERR induction have been proposed by Meng and coworkers [68]. The scaffold is a MOF that contains a disulfide imidazole ligand coordinated with zinc and encapsulating Ce6 (Ce6@RMOF, Table 4). Following irradiation (660 nm), the NPs promote FERR through disulfide-thiol exchange, which depletes GSH levels, thereby attenuating GPX4 activity and increasing lipid peroxides. These findings have been observed in vitro and in vivo in mouse mammary 4T1 tumor. The tumor growth inhibition and the reduced GPX4 activity are counteracted by the co-administration of iron chelators (i.e., FERR inhibitor).

NPs (SRF@FeIIITA) based on Fe^3+^ and TA that form a network shell encapsulating SFB have been reported by Liu et al. (Table 4) [69]. Following cellular uptake and irradiation (660 nm), the NPs accumulate into the lysosomes where the acidic environment allows their dissolution, in turn, favoring Fe^3+^/Fe^2+^ reduction and SFB release. SFB reduces GSH levels and inhibits GPX4 activity, which improves the FERR induced by Fe^2+^ (e.g., lipid peroxide augmentation). A reduced cell proliferation was observed in 4T1 cells exposed to SRF@FeIIITA upon 660 nm irradiation. The in vitro results have been corroborated by the efficient inhibition of tumor growth observed in vivo in mice bearing 4T1 tumors treated with SRF@FeIIITA.

## 4. Conclusions

FERR is a recently reported mechanism of regulated cell death under intensive investigation, and numerous small molecules that induce FERR are emerging. However, in spite of the interesting activity in overcoming the resistance to apoptosis developed by tumors exposed to conventional chemotherapeutics, FERR inducers show drawbacks typical of small molecules including low solubility, limited tumor targeting, and toxic side effects that have hampered their clinical evaluation. Nanomedicine has proven efficacy in overcoming the numerous drawbacks manifested by the small molecules, and NPs engineered for inducing FERR are now implementing the armamentarium available to date for fighting tumors. Such NPs improve the circulation time of loaded drugs as well as tumor targeting. Generally, these NPs show tumor selectivity via the EPR effect and, due to their loading capability, release cargo and stimulate anticancer immunity at the tumor site. The particle nature of NPs allows for their specific decoration for the targeting of peculiar tumor molecular properties or physiologic conditions (active targeting), thus potentiating the EPR effect (passive targeting). Therefore, their loading capability also holds promise for fighting multidrug resistant tumors. Indeed, besides inducing FERR, the NPs are designed to release drugs that hit different deregulated pathways of cancer cells. Additionally, some NPs show magnetic properties, allowing for the exploitation of the magnetic field for MRI as well as magnetic-guided drug delivery at the tumor site (theranostics). In this context, NPs projected for improving the potency of novel medical approaches in the use for cancer management including RFA, hyperthermia, and PDT appear to be very interesting. Of note, almost all the NPs inducing FERR have been tested in vivo and showed a favorable antitumor profile with no important side effects. However, as in the case of small molecules inducing FERR, no clinical trial containing the reported NPs is ongoing.

In summary, to ultimately demonstrate the safety profile as well as their effectiveness as antitumor targeted therapeutics, clinical investigations are mandatory. This implies that, despite the preclinical success achieved by these NPs, additional efforts are required in the future.

## Figures and Tables

**Table 1:** Nanoparticles for antitumor drug delivery.

Nanoparticle	Drug Loaded	Tumor Type
DAR	Doxorubicin	Human MCF7 breast cancer Mouse mammary 4T1 tumor
ACC@DOX–CaSi–PAMAM–FA/mPEG	Doxorubicin	Mouse mammary 4T1 tumor Human A375 melanoma
DGU:Fe	Doxorubicin	Human MCF7 breast cancer Mouse mammary 4T1 tumor
PtH@FeP	Cisplatin	Human HepG2 hepatocellular carcinoma Human A2780 ovarian cancer
FeGd-HN@Pt@LF/RGD2	Cisplatin	Human U-87 MG glioblastoma
UCNP@LP(Azo-CA4)	Azobenzene combretastatin A4	Human MDA-MB-231 breast cancer

**Table 2:** Nanoparticles for tumor targeting.

Nanoparticle	Tumor Targeting Mechanism	Tumor Type
Tf-LipoMof@PL	Trasferrin	Mouse mammary 4T1 tumor
Malt-PEG-Abz@RSL3	GLUT	Human HepG2 hepatocellular carcinoma
NMIL-100@GOx@C	Starvation and glucose oxidase	Mouse mammary 4T1 tumor
AMSNs	Arginine transporter	Human Huh 7 hepatocellular carcinoma
API-Fe_2_O_3_/Fe_3_O_4_@mSiO_2_-HA	Hyaluronic acid and magnetic field	Human A549 lung carcinoma
mPEG-Azo-PBLA@RSL3	Hypoxia	Mouse mammary 4T1 tumor
FaPEG-MnMSN@SFB	Hyperoxidative condition	Human HepG2 hepatocellular carcinoma Mouse mammary 4T1 tumor Human A549 lung carcinoma
RSL3@COF-Fc	Hyperoxidative condition	Human HT1080 fibrosarcoma Human MCF7 breast cancer Human HCT116 colon carcinoma
CSO-SS-Cy7-Hex/SPION/Srfn	Hyperoxidative condition	Mouse mammary 4T1 tumor Human MDA-MB-231 breast cancer
TA-Fe/ART@ZIF	pH responsive	Human MDA-MB-231 breast cancer
HA@MOF	pH responsive	Mouse mammary 4T1 tumor
WA-NPs	pH responsive	Human IMR-32 neuroblastoma
Pyrite-peroxidase nanozyme	Folate/folate receptor	Mouse CT26 colon adenocarcinoma
PSAF NCs	pH responsive	Mouse mammary 4T1 tumor

**Table 3:** Nanoparticles for immunomodulation.

Nanoparticle	Mechanism of Action	Tumor Type
MIL88B/RSL3	M1 polarization of TAMs	Mouse mammary 4T1 tumor
ZVI-NP	M1 polarization of TAMs CD8+T cell increase Regulatory T cells reduction	Human A549 lung carcinoma Human H460 lung carcinoma Human HT1299 lung carcinoma Mouse LLC lung carcinoma
Pa-M/Ti-NC	M1 polarization of TAMs Generation of an immunogenic microenvironment that favors ferroptosis	Mouse mammary 4T1 tumor

**Table 4:** Nanoparticles for radiofrequency ablation, hyperthermia, and photodynamic therapy.

Nanoparticle	Mechanism of Action	Tumor Type
HLCaP	Radiofrequency ablation	Mouse mammary 4T1 tumor Mouse H22 hepatocellular carcinoma Rabbit VX2 hepatocellular carcinoma
GBP@Fe_3_O_4_	Hyperthermia	Human MBA-MD-231 breast cancer Human 786-O renal cell carcinoma Human PC3 prostate cancer Human C4-2 prostate cancer
Fe_3_O_4_-PLGA-Ce6	Photodynamic therapy	Mouse mammary 4T1 tumor
PEG-Fns	Photodynamic therapy	Mouse mammary 4T1 tumor
PB@FePt-HA-g-PEG	Photodynamic therapy	Mouse mammary 4T1 tumor Human MCF7 breast cancer
SRF@Hb-Ce6	Photodynamic therapy	Mouse mammary 4T1 tumor Human A549 lung carcinoma Human HepG2 hepatocellular carcinoma
Ce6@RMOF	Photodynamic therapy	Mouse mammary 4T1 tumor
SRF@Fe^III^TA	Photodynamic therapy	Mouse mammary 4T1 tumor

## Data Availability

Not applicable.
